# Tumour-infiltrating lymphocytes as a prognostic and tamoxifen predictive marker in premenopausal breast cancer: data from a randomised trial with long-term follow-up

**DOI:** 10.1186/s13058-020-01364-w

**Published:** 2020-12-23

**Authors:** Christine Lundgren, Pär-Ola Bendahl, Maria Ekholm, Mårten Fernö, Carina Forsare, Ute Krüger, Bo Nordenskjöld, Olle Stål, Lisa Rydén

**Affiliations:** 1 Department of Oncology, Region Jönköping County, Jönköping, Sweden; 2 Department of Clinical Sciences Lund, Division of Oncology, Lund University, Lund, Sweden; 3 Department of Biomedical and Clinical Sciences, Linköping University, Linköping, Sweden; 4 Department of Clinical Sciences Lund, Division of Surgery, Lund University, Lund, Sweden; 5 Department of Surgery, Skåne University Hospital, Malmö, Sweden

**Keywords:** Breast cancer, TILs, Prognosis, Tamoxifen, Premenopausal, Predictive, Biomarker

## Abstract

**Background:**

Tumour-infiltrating lymphocytes (TILs) are of important prognostic and predictive value in human epidermal growth factor receptor 2-positive (HER2+) breast cancer (BC) and triple-negative breast cancer (TNBC), but their clinical relevance in oestrogen receptor-positive/HER2-negative (ER+/HER2−) remains unknown. The primary study aim was to analyse the prognostic effect of TILs on the BC-free interval (BCFi) in premenopausal patients stratified by BC subtypes. The secondary aim was to investigate if TILs are predictive of tamoxifen (TAM) benefit.

**Methods:**

Archival tissues from primary breast tumours were collected from patients from the SBII:2pre trial, in which 564 premenopausal women were randomised to 2 years of adjuvant TAM or no systemic treatment, regardless of hormone receptor status. TILs were scored on whole tissue sections from 447 patients with available ER status. Tumours were divided into ER+/HER2−, HER2+ and TNBC subtypes by immunohistochemistry and in situ hybridisation. The prognostic value of TILs was analysed in systemically untreated patients (*n* = 221); the predictive information was investigated in the ER+ subgroup (*n* = 321) by cumulative incidence curves and Cox regression analyses. The median follow-up was 28 years.

**Results:**

High (≥ 50%) infiltration of TILs was a favourable prognostic factor in terms of BCFi (univariable analysis: hazard ratio_BCFi_ (HR_BCFi_) 0.40; 95% confidence interval (CI) 0.22–0.71; *P* = 0.002). Similar effects were observed across all BC subtypes. The effect of adjuvant TAM was stronger in patients with ER+ tumours and TILs < 50% (HR_BCFi_ 0.63; 95% CI 0.47–0.84; *P* = 0.002) than in patients with high immune infiltration (≥ 50%) (HR_BCFi_ 0.84; 95% CI (0.24–2.86); *P* = 0.77). However, evidence for differential effects of TAM in categories of TILs, i.e. interaction, was weak.

**Conclusions:**

We demonstrate a long-term favourable prognostic value of high infiltration of TILs in a cohort of premenopausal BC patients and the positive prognostic effect was extended to the ER+/HER2− subgroup. A beneficial effect of TAM in ER+ patients was observed in patients with tumours of low TIL infiltration, but evidence for a treatment predictive effect was weak.

**Trial registration:**

This trial is registered in the ISRCTN database, trial ID: ISRCTN12474687.

**Supplementary information:**

The online version contains supplementary material available at 10.1186/s13058-020-01364-w.

## Background

The breast cancer (BC) subtypes, as determined by either gene expression analysis or surrogate immunohistochemical (IHC) markers (oestrogen receptor (ER), progesterone receptor (PR), human epidermal growth factor receptor 2 (HER2), proliferation marker (Ki67), and Nottingham histological grade (NHG), have different prognostic and predictive characteristics [1, 2]. In addition, they show differences in immune biology and mutational load; for example, the HER2-positive (HER2+) BC and triple-negative breast cancer (TNBC) subtypes have a higher lymphocyte infiltration compared with ER-positive/HER2-negative (ER+/HER2−) tumours [3] and moreover, a higher mutational load is observed in ER-negative (ER–) tumours than ER+ tumours [4].

Lymphocyte-predominant BC (LPBC) are tumours with a higher proportion of immune cell infiltration than invasive tumour cells [5]. An abundance of tumour-infiltrating lymphocytes (TILs) has been shown to indicate good prognosis, particularly for the HER2+ and TNBC subtypes [6–9]. In HER2+ tumours, high level of TILs has been linked to different responsiveness to chemotherapy and increased efficacy of adjuvant trastuzumab treatment [3, 7, 10]. However, data on the influence of lymphocyte infiltration on prognoses and therapy prediction in patients with ER+/HER2− tumours are sparse [3, 6, 11].

In TNBC and HER2+ BC, LPBC predict pathological complete response (pCR) after neoadjuvant chemotherapy [6, 12]. This association has also been reported in the hormone receptor-positive /HER2− subgroup, in which a high level of TILs is associated with pCR [6, 13]. However, results regarding the ability of TILs to predict the effect of neoadjuvant endocrine therapy are sparse [14, 15]. In addition, except for some studies of TIL phenotypes, there are no reports on TILs as predictors for adjuvant endocrine therapy in the ER+/HER2− subgroup.

Despite the beneficial effects of adjuvant tamoxifen (TAM) in ER+ BC [16], some patients experience late recurrences after diagnosis [17]. Currently, no markers have been identified that predict late BC-related events, and any potential predictors must be investigated in trials with long-term follow up. The SBII:2pre randomised controlled trial included premenopausal women that received 2 years of adjuvant TAM or no adjuvant systemic therapy during 1984–1991, irrespective of hormone receptor status. We have previously reported the beneficial effect of 2 years of TAM in this trial based on long-term (~ 30 years) outcome [18]. This cohort provides an excellent basis for further studies on the prognostic effect and predictive value of TILs in relation to adjuvant TAM therapy in premenopausal patients for whom TAM is still a commonly recommended endocrine therapy [19, 20].

The primary aim of this study was to investigate the prognostic value of high infiltration of TILs in premenopausal patients across different BC subtypes including ER+/HER2− BC from the SBII:2pre randomised controlled trial. Our secondary aim was to investigate TILs as a predictive marker for TAM efficacy in the ER+ subgroup.

## Methods

### Patients and study cohorts

**Fig. 1:**
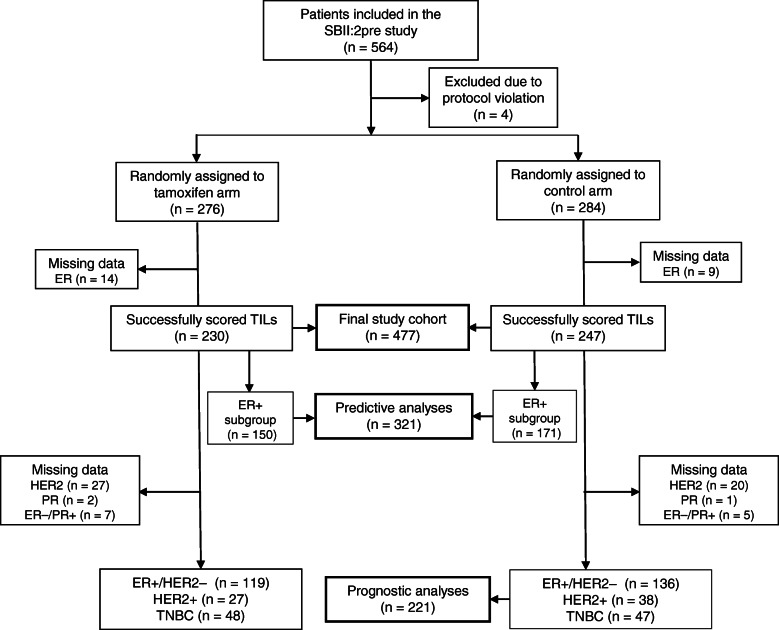
Flow chart of the study cohort. *Abbreviations:* *ER* oestrogen receptor, *HER2* human epidermal growth factor receptor 2, *PR* progesterone receptor, *TILs* tumour-infiltrating lymphocytes, *TNBC* triple-negative breast cancer

The patients in this study participated in the SBII:2pre study and details of the study have been previously presented [18, 21, 22]. Briefly, during 1984–1991, 564 premenopausal women with stage II invasive BC (UICC TNM, third edition (1982)) were randomised between 2 years of adjuvant TAM or no systemic treatment. Two coordinating centres including 20 hospitals participated in the study: the South Eastern (Oncological Centre Lund) and Southern (Oncological Centre Linköping) Health Care Regions. Four patients were excluded in the latest update of the study due to protocol violations found by scrutiny of the patient records [18]. Among the included 560 patients, 284 were randomised to the control arm and 276 to the TAM treatment arm (Fig. 1). In the present study, the prognostic value of TILs was evaluated in patients allocated to no systemic therapy with tumours successfully scored for TILs and available IHC/in situ hybridisation (ISH) data for defining BC subtypes. All patients with ER+ tumours and successfully annotated TILs were included in the assessment of the prediction of TAM efficacy (Fig. 1).

### Follow-up data

Data on invasive distant, regional and local recurrence, contralateral BC, BC-related death and death due to other causes were determined based on a thorough review of all medical records and the Swedish Cause of Death Register as previously described [18].

### Tumour characteristics and microscopic assessments of TILs and lymphovascular invasion (LVI)

Tissue microarrays were used for assessment of ER, PR, Ki67 and HER2. ER and PR were assessed by IHC and ER/PR-positivity was defined as tumours with > 10% stained nuclei according to Swedish Guidelines [23]. Data on both IHC and the cytosol-based method were available; for tumours with missing IHC data, the results from the original cytosol-based methods were used (ER: *n* = 32; PR: *n* = 46). Ki67 was assessed as a categorical variable (≤ 10%, 11–25%, ≥ 26%) [24]. Tumours were classified as HER2+ either by *HER2* amplification by fluorescent ISH (*n* = 54) or by HER2 3+ as assessed by IHC in cases in which ISH data were missing (*n* = 12)*.* Histological grade was evaluated as NHG according to Elston et al. [25]. The tumours were stratified into three subtypes based on IHC and ISH markers: ER+/HER2−, HER2+ (irrespective of ER status) and TNBC (ER−/PR−/HER2−).

**Fig. 2:**
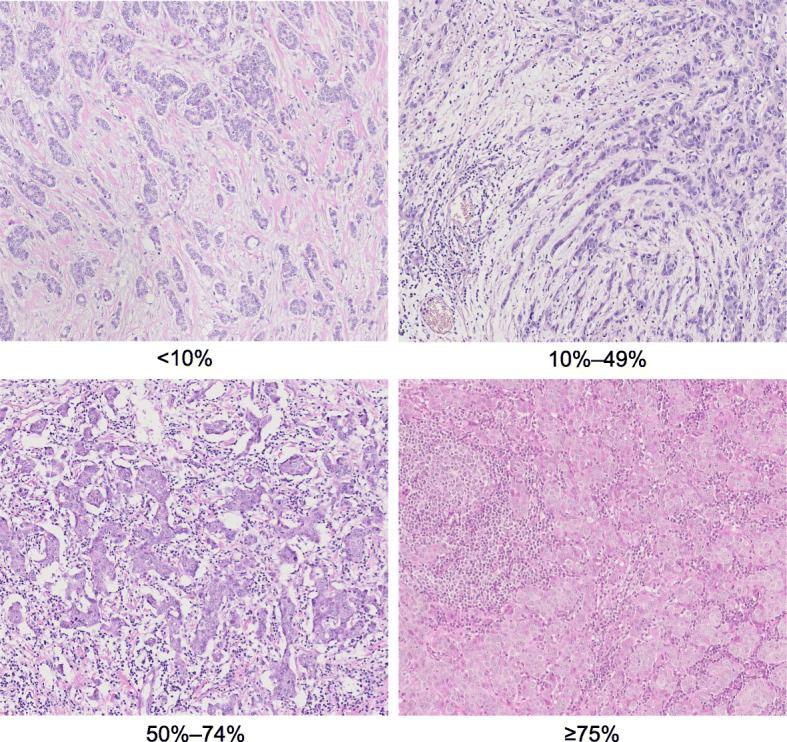
Variable degree of lymphocytic infiltration on haematoxylin and eosin-stained tumour sections (× 200 magnification)

Archival formalin-fixed paraffin-embedded tissues from breast tumours in the SBII:2pre trial were collected from seven regional biobanks and stained by haematoxylin-eosin (*n* = 520). Of these, 488 were available for TIL scoring and 486 for assessment of LVI. The scoring of TILs was performed according to the definition by the Immuno-Oncology International TILs Working Group, in which stromal TILs (referred to as TILs in the current study) are defined as the proportion of the stromal area containing infiltration of lymphocytes with no direct contact with invasive tumour cells [5]. Microscopic assessment was performed by a board-certified breast pathologist (Ute Krüger) blinded to the patient characteristics and outcomes. TILs were assessed under a light microscope (BX63F, Olympus, Japan) with a magnification of 40 and 100 (if necessary, 200). Tumours were categorised into the following groups based on TIL infiltration: < 10%, 10–49%, 50–74% and ≥ 75%. In the prognostic analyses, the two latter groups were merged into one category (high), and the three groups were then denoted as low, intermediate and high. Tumours with TILs ≥ 50% were also defined as LPBC and tumours with low/intermediate TILs (< 50%) were defined as non-LPBC in the predictive analyses. Photomicrographs of the different TIL categories are shown in Fig. 2.

According to the Swedish pathological guidelines, LVI was defined as present when tumour cells in cavities lined with endothelium (not by IHC endothelial markers) were verified in the peritumour area [23]. Patient and tumour characteristics have been reported previously [18, 21, 22] and are listed in Additional file 1, stratified by tumours with and without scored TILs.

### Statistical analyses

Differences in distribution between clinico-pathological variables and TILs were analysed by chi2 test and chi2 test for trend. The primary endpoint was BC-free interval (BCFi), defined as the first event of local, regional or distant recurrence, contralateral BC (invasive or ductal cancer in situ (DCIS)) or BC-related death. The association with overall survival (OS) was also explored. The data cut-off date for events was November 30, 2016.

The cumulative incidence of BC-related events as a function of follow-up time was estimated and compared among patient subgroups to handle the problem with competing risks. To facilitate the comparison of results for the two endpoints, BCFi and OS, cumulative incidence, which is the same as one minus the Kaplan–Meier estimate, was also used for the endpoint death from all causes. The log-rank test was used to evaluate the evidence for difference between cumulative incidence curves and the trend version of the test was used for comparison with more than two ordered groups. Hazard ratios (HR) were calculated by Cox regression analyses, stratified by region.

The follow-up was censored in the analysis of BCFi if a patient died from a cause that was not BC-related without a preceding BC event included in the definition of an event for BCFi. Hence, the estimated HRs for BCFi in this cause-specific Cox regression analysis should be interpreted in an imaginary world where all other causes of death have been eliminated. In the predictive multivariable analyses, PR status was omitted due to collinearity with ER status. A Cox model with a term for interaction between TIL subgroup (LPBC vs. non-LPBC) and TAM was fitted to evaluate the evidence for differential effect of TAM in the two TIL subgroups. Exclusion of the seven cases with mostly DCIS in addition to microinvasion did not change the results, and these were therefore included in the analyses.

HRs are presented with 95% confidence intervals (CIs). All statistical tests are two-sided. The statistical calculations were performed with IBM SPSS Statistics, Version 25.0 (IBM Corp., Armonk, NY, USA), and the cumulative incidence curves were drawn using STATA, Version 16.1 (StataCorp LLC, College Station, TX, USA).

## Results

Tumours were successfully scored for TILs into the following groups: low (< 10%), intermediate (10–49%) and high (≥ 50%) groups. For tumours with available ER status (*n* = 477), approximately half of the tumours were in the low group (52%, 248/477), while 33% (157/477) were in the intermediate group and 15% (72/477) were in the high group. There were a total of 321 ER+ tumours and 320 PR+ tumours. After exclusion of tumours with missing HER2 and/or PR status as well as ER−/PR+ tumours (considered an inconclusive subgroup [26, 27]), the tumours were classified as follows: ER+/HER2−, 61% (*n* = 255/415); HER2+, 16% (*n* = 65/415); and TNBC, 23% (*n* = 95/415) (Fig. 1). There were 153 BC-related events in the control-arm during the follow-up. The number of BC-related events in the control and TAM group for prediction analyses (ER+ tumours) were 119 and 81, respectively. The median follow-up for patients without any BC-related events was 28 years.

### Distribution of TILs in relation to clinicopathological variables

The distribution of TILs in relation to patient and tumour characteristics in the study cohort (*n* = 477) is presented in Table 1. A high proportion of immune cell infiltration was associated with younger age, high histological grade, ER−, PR− and HER2+ status, high Ki67 and medullary histological type. None of the lobular tumours had high infiltration of TILs. The frequency of high TILs in ER+/HER2−, HER2+ and TNBC subgroups was 6% (*n* = 16/257), 24% (*n* = 16/66) and 35% (*n* = 33/95), respectively. The frequency of low TILs was 69% (*n* = 176/257), 33% (*n* = 22/66) and 21% (*n* = 20/95), respectively.

**Table 1:** Distribution of TILs according to patient and tumour characteristics (*n* = 477)

Variable	TIL low (< 10%) ***n*** (%)	TIL intermediate (10–49%) ***n*** (%)	TIL high (≥ 50%) ***n*** (%)	***P*** value^**a**^
Age (years)				0.02
< 40	41 (44)	32 (34)	21 (22)	
≥ 40	207 (54)	125 (33)	51 (13)	
Nodal status				0.34
0	65 (47)	42 (31)	30 (22)	
1–3	137 (59)	66 (28)	30 (13)	
≥ 4	46 (44)	47 (45)	12 (11)	
Missing	0	2	0	
Tumour size (mm)				0.06
≤ 20	97 (57)	52 (31)	20 (12)	
> 20	151 (49)	104 (34)	52 (17)	
Missing	0	1	0	
Histological grade (NHG)				< 0.001
1	44 (86)	7 (14)	0	
2	135 (70)	52 (27)	5 (3)	
3	56 (27)	91 (43)	64 (30)	
Missing	13	7	3	
ER				< 0.001
Negative	45 (29)	63 (40)	48 (31)	
Positive	203 (63)	94 (29)	24 (8)	
PR				< 0.001
Negative	41 (27)	63 (41)	50 (33)	
Positive	206 (64)	93 (29)	21 (7)	
Missing	1	1	1	
HER2				0.001
Negative	205 (56)	109 (30)	50 (14)	
Positive	22 (33)	28 (42)	16 (24)	
Missing	21	20	6	
LVI				0.87
Absent	140 (54)	76 (30)	42 (16)	
Present	108 (50)	81 (37)	28 (13)	
Missing	0	0	2	
Ki67 (%)				< 0.001
≤ 10	126 (71)	40 (23)	12 (7)	
11–25	60 (56)	33 (31)	14 (13)	
≥ 26	22 (20)	53 (48)	36 (32)	
Missing	40	31	10	
Histopathological type				< 0.001
Ductal/NST	200 (52)	134 (35)	48 (13)	
Lobular	29 (81)	7 (19)	0	
Medullary	0	3 (13)	20 (87)	
Other	9 (75)	2 (17)	1 (8)	
Missing	10	11	3	
Subtype				< 0.001
ER+/HER2−	176 (69)	65 (25)	16 (6)	
HER2+	22 (33)	28 (42)	16 (24)	
TNBC	20 (21)	42 (44)	33 (35)	
Missing	30	22	7	
**Total**	248 (52)	157 (33)	72 (15)	

^a^ Chi2 test for trend, except for the non-ordinal variables histopathological type and subtype, when conventional chi2 test was used

*Abbreviations*: *ER* oestrogen receptor, *HER2* human epidermal growth factor receptor 2, *LVI* lymphovascular invasion, *NST* no special type, *NHG* Nottingham histological grade, *PR* progesterone receptor, *TILs* tumour-infiltrating lymphocytes, *TNBC* triple-negative breast cancer

### TILs as a prognostic marker for BC events

**Fig. 3:**
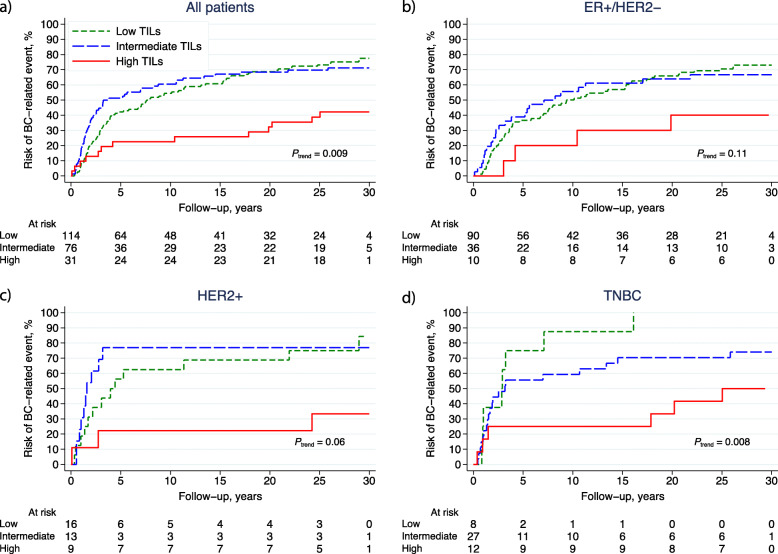
**a**–**d** Cumulative incidence of breast cancer (BC)-related events (BCFi) in different breast cancer subtypes. The panel illustrates the result of different levels of TILs in **a** all patients, and patients with the following breast cancer subtypes; **b** ER+/HER2−; **c** HER2+; and **d** TNBC. The patients were allocated to no adjuvant systemic treatment and TILs were categorised as low: < 10%, intermediate: 10–49% and high: ≥ 50%. *Abbreviations:* *BCFi* breast cancer-free interval, *ER* oestrogen receptor, *HER2* human epidermal growth factor receptor 2, *TILs* tumour-infiltrating lymphocytes, *TNBC* triple-negative breast cancer

**Fig. 4:**
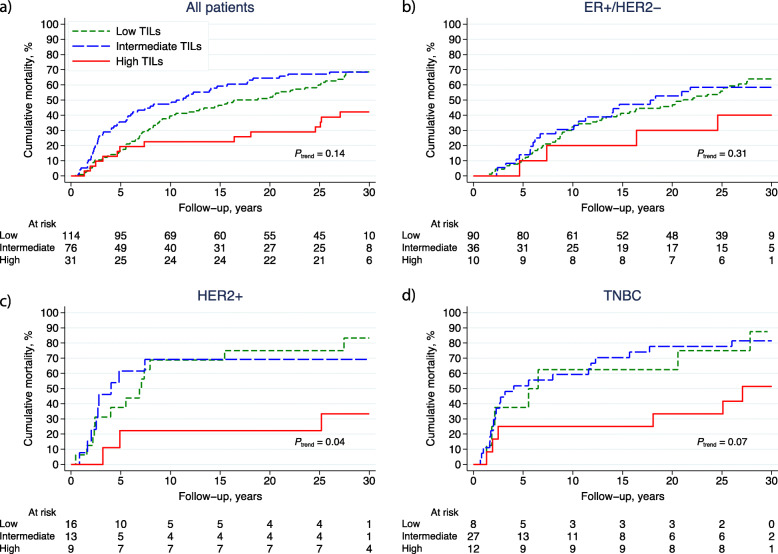
**a**–**d** Cumulative mortality (OS) in different breast cancer subtypes. The panel illustrates the result of different levels of TILs in **a** all patients, and patients with the following breast cancer subtypes; **b** ER+/HER2−; **c** HER2+; and **d** TNBC. The patients were allocated to no adjuvant systemic treatment and TILs were categorised as low: < 10%, intermediate: 10–49% and high: ≥ 50%. *Abbreviations:* *ER* oestrogen receptor, *HER2* human epidermal growth factor receptor 2, *OS* overall survival, *TILs* tumour-infiltrating lymphocytes, *TNBC* triple-negative breast cancer

The prognoses in terms of BCFi and OS stratified by the three TIL categories (low, intermediate, high) are displayed in Figs. 3a–d and 4a–d, respectively. The prognostic value of TILs was evaluated for all patients included in the control arm (*n* = 221) stratified by subtypes (ER+/HER2−, *n* = 136; HER2+, *n* = 38; and TNBC, *n* = 47) (Fig. 1). All patients with high TILs, irrespective of BC subtype, had improved prognosis compared with patients with low TILs (HR_BCFi_ 0.40; 95% CI 0.22–0.71; *P* = 0.002, and HR_OS_ 0.52; 95% CI 0.29–0.95; *P* = 0.03) (Table 2). This was true also in multivariable analysis adjusting for age, nodal status, tumour size, histological grade, ER, PR, HER2 and LVI (HR_BCFi_ 0.22; 95% CI 0.11–0.43; *P* < 0.001 and HR_OS_ 0.23; 95% CI 0.11–0.48; *P* < 0.001). The univariable prognostic effect of high vs. low TILs was essentially the same in patients with ER+/HER2− tumours (HR_BCFi_ 0.40; 95% CI 0.14–1.09; *P* = 0.07) as well as in HER2+ (HR_BCFi_ 0.28; 95% CI 0.06–0.97; *P* = 0.05) and TNBC tumours (HR_BCFi_ 0.27; 95% CI 0.08–0.88; *P* = 0.03). The prognostic effect of high TILs was also observed in the multivariable analysis, except for patients with TNBC (Table 2). Presence of LVI was associated with a worse prognosis in patients in the control arm in the univariable analysis (HR_BCFi_ 1.49; 95% CI 1.08–2.05; *P* = 0.02), and the results were essentially the same in multivariable analysis (HR_BCFi_ 1.39; 95% CI 0.99–1.95; *P* = 0.06) (Table 2).

**Table 2:** Cox regression analyses of BCFi and OS in patients randomised to no adjuvant medical treatment

	Univariable		Multivariable^**a**^	
Variable	BCFi	OS	BCFi	OS
	HR (95% CI); ***P*** value			
**TILs, category**^**b**^				
**All subtypes**	(*n* = 221)		(*n* = 213)	
Low (Ref.)	1.00	1.00	1.00	1.00
Intermediate	1.10 (0.78–1.54); 0.61	1.26 (0.88–1.80); 0.21	0.61 (0.40–0.93); 0.02^c^	0.65 (0.41–1.02); 0.06
High	0.40 (0.22–0.71); 0.002	0.52 (0.29–0.95); 0.03	0.22 (0.11–0.43); < 0.001	0.23 (0.11–0.48); < 0.001
**ER+/HER2−**	(*n* = 136)		(*n* = 135)	
Low (Ref.)	1.00	1.00	1.00	1.00
Intermediate	1.02 (0.63–1.64); 0.94	1.02 (0.61–1.71); 0.95	0.69 (0.42–1.15); 0.16	0.65 (0.37–1.15); 0.14
High	0.40 (0.14–1.09); 0.07	0.55 (0.20–1.52); 0.25	0.20 (0.06–0.60); 0.004	0.30 (0.10–0.96); 0.04
**HER2+**	(*n* = 38)		(*n* = 35)	
Low (Ref.)	1.00	1.00	1.00	1.00
Intermediate	1.47 (0.62–3.49); 0.39	1.07 (0.45–2.56); 0.88	0.47 (0.14–1.60); 0.23	0.38 (0.11–1.31); 0.13
High	0.28 (0.08–0.97); 0.05	0.27 (0.08–0.96); 0.04	0.06 (0.01–0.56); 0.01	0.05 (0.01–0.39); 0.005
**TNBC**	(*n* = 47)		(*n* = 43)	
Low (Ref.)	1.00	1.00	1.00	1.00
Intermediate	0.76 (0.31–1.87); 0.55	1.24 (0.49–3.14); 0.65	0.59 (0.21–1.67); 0.32	1.02 (0.34–3.11); 0.97
High	0.27 (0.08–0.88); 0.03	0.44 (0.14–1.36); 0.16	0.38 (0.11–1.39); 0.15	0.59 (0.16–2.26); 0.44
**Covariables**	(*n* = 216–221)		(*n* = 213)	
**Age (years)**				
< 40 (Ref.)	1.00	1.00	1.00	1.00
≥ 40	0.72 (0.49–1.04); 0.08	0.65 (0.44–0.96); 0.03	0.60 (0.40–0.89); 0.01	0.56 (0.37–0.85); 0.006
**Nodal status**				
0 (Ref.)	1.00	1.00	1.00	1.00
1–3	1.31 (0.87–1.96); 0.19	1.59 (1.03–2.47); 0.04	1.21 (0.77–1.91); 0.40	1.69 (1.03–2.77); 0.04
≥ 4	2.33 (1.49–3.64); < 0.001	2.81 (1.75–4.52); < 0.001	2.03 (1.25–3.29); 0.004	2.70 (1.58–4.52); < 0.001
**Tumour size (mm)**				
≤ 20 (Ref.)	1.00	1.00	1.00	1.00
> 20	1.04 (0.75–1.45); 0.80	0.92 (0.66–1.29); 0.63	1.23 (0.86–1.76); 0.25	1.04 (0.72–1.51); 0.84
**Histological grade (NHG)**				
1 (Ref.)	1.00	1.00	1.00	1.00
2	1.51 (0.84–2.72); 0.17	1.34 (0.73–2.45); 0.35	1.36 (0.74–2.48); 0.32	1.13 (0.60–2.11); 0.71
3	1.82 (1.03–3.23); 0.04	1.96 (1.09–3.54); 0.03	2.70 (1.36–5.33); 0.004	2.25 (1.08–4.66); 0.03
**ER**				
Negative (Ref.)	1.00	1.00	1.00	1.00
Positive	0.94 (0.66–1.34); 0.74	0.69 (0.49–0.99); 0.04	0.41 (0.12–1.36); 0.15	0.37 (0.11–1.27); 0.11
**PR**				
Negative (Ref.)	1.00	1.00	1.00	1.00
Positive	1.06 (0.75–1.49); 0.75	0.80 (0.57–1.14); 0.21	2.38 (0.77–7.30); 0.13	1.76 (0.57–5.48); 0.33
**HER2**				
Negative (Ref.)	1.00	1.00	1.00	1.00
Positive	1.10 (0.72–1.69); 0.65	1.26 (0.81–1.94); 0.30	1.05 (0.66–1.67); 0.85	1.11 (0.68–1.82); 0.67
**LVI**				
Absent (Ref.)	1.00	1.00	1.00	1.00
Present	1.49 (1.08–2.05); 0.02	1.26 (0.90–1.76); 0.18	1.39 (0.99–1.95); 0.06	1.05 (0.73–1.51); 0.78

All analyses were stratified by study region

*Abbreviations*: *BCFi* breast cancer free-interval, *CI* confidence interval, *ER* oestrogen receptor, *HER2* human epidermal growth factor receptor 2, *HR* hazard ratio, *LVI* lymphovascular invasion, *NHG* Nottingham histological grade, *OS* overall survival, *PR* progesterone receptor, *TILs* tumour-infiltrating lymphocytes, *TNBC* triple-negative breast cancer

^a^The following variables were included in multivariable analysis: age (≥ 40 vs. < 40 years), nodal status (0 vs.1–3 vs. ≥ 4), tumour size (> 20 mm vs. ≤ 20 mm), histological grade (1 vs. 2 vs. 3), ER (positive vs. negative), PR (positive vs. negative), HER2 (positive vs. negative), LVI (present vs. absent) and TILs (high vs. intermediate vs. low)

^b^TILs were categorised as low: < 10%, intermediate: 10–49% and high: ≥ 50%

^c^A series of multivariable analyses including TILs and only one additional prognostic variable at a time revealed that the univariable effect of intermediate vs. low TILs on outcome was mainly confounded by NHG. In the prognostic cohort, 65% of the patients with intermediate TILs had NHG 3 tumours

### TILs as a predictive marker for TAM benefit in the ER+ subgroup

**Fig. 5:**
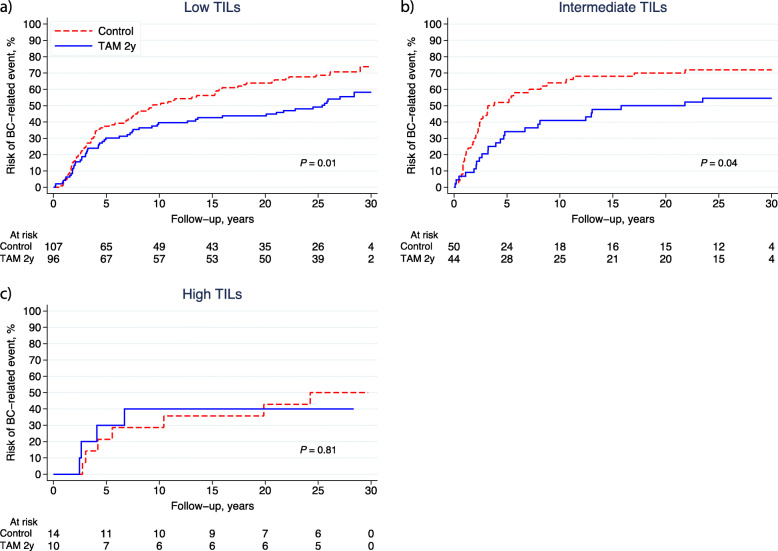
**a**–**c** Cumulative incidence of breast cancer (BC)-related events (BCFi) stratified by treatment allocation (control vs. TAM) for patients whose tumours were ER-positive and had **a** low TILs (< 10%); **b** intermediate TILs (10–49%) and **c** high TILs (≥ 50%). *Abbreviations:* *BCFi* breast cancer-free interval, *ER* oestrogen receptor, *TAM* tamoxifen, *TILs* tumour-infiltrating lymphocytes

**Fig. 6:**
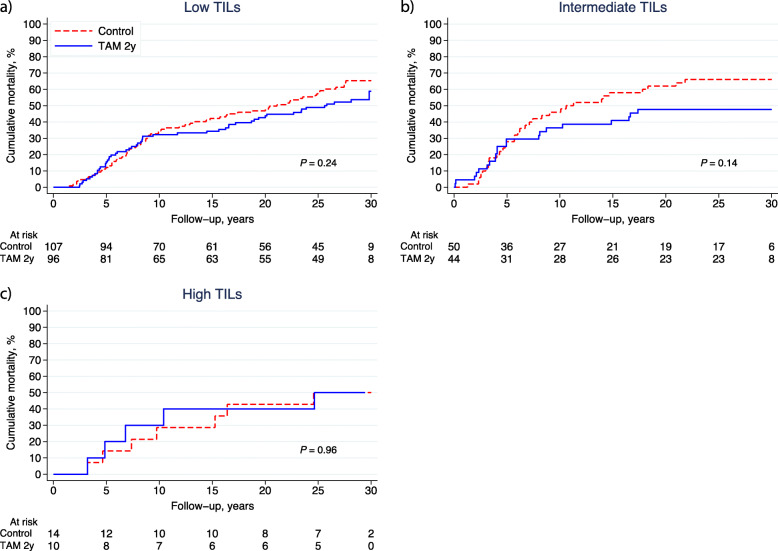
**a**–**c** Cumulative mortality (OS) stratified by treatment allocation (control vs. TAM) for patients whose tumours were ER-positive and had **a** low TILs (< 10%); **b** intermediate TILs (10–49%) and **c** high TILs (≥ 50%). *Abbreviations:* *ER* oestrogen receptor, *OS* overall survival, *TAM* tamoxifen, *TILs* tumour-infiltrating lymphocytes

The predictive value of TILs for TAM treatment was evaluated in the ER+ cohort (*n* = 321). In our previous follow-up study, TAM prolonged BCFi in the study population with ER+ tumours (*n* = 362) (HR_BCFi_ 0.62; 95% CI 0.47–0.82; *P* = 0.001) [18], and this was also true in the present study cohort (*n* = 321) (HR_BCFi_ 0.65; 95% CI 0.49–0.86; *P* = 0.002). The proportions of tumours with low, intermediate and high TILs were 63% (*n* = 107/171), 29% (*n* = 50/171) and 8% (*n* = 14/171) in the control group and 64% (*n* = 96/150), 29% (*n* = 44/150) and 7% (*n* = 10/150) in the TAM group, respectively. Figure 5a–c illustrates the outcome (BCFi) stratified by TIL categories and treatment allocation. In the univariable analysis, TAM improved the outcome for patients with low (HR_BCFi_ 0.66; 95% CI 0.46–0.93; *P* = 0.02) and intermediate TILs (HR_BCFi_ 0.59; 95% CI 0.35–1.00, *P* = 0.05). In contrast, the outcome of patients with high TILs was not affected by adjuvant TAM (HR_BCFi_ 0.89; 95% CI 0.26–3.07; *P* = 0.86). There was no clear association between OS and TIL categories as illustrated in Fig. 6a–c.

Furthermore, the differential effect of TAM treatment on BCFi in TIL subgroups was analysed in a Cox model including TAM treatment and TILs and an interaction term. The TIL variable was divided into two categories at an exploratory 50% cut-off (non-LPBC as < 50% vs. LPBC as ≥ 50%), based on the above predictive results. Among the ER+ samples, 93% (*n* = 297/321) were categorised as non-LPBC and 7% (*n* = 24/321) as LPBC. The effect of TAM was stronger in patients with non-LPBC (HR_BCFi_ 0.63; 95% CI 0.47–0.84; *P* = 0.002) than in patients with LPBC (HR_BCFi_ 0.84; 95% CI 0.24–2.86; *P* = 0.77), but the evidence for an interaction between TAM treatment and level of TIL infiltration on BCFi was weak (*P*_interaction_ = 0.65) (Table 3).

**Table 3:** Predictive value of TILs for TAM response with respect to breast cancer-free interval (ER-positive cohort)

	Univariable (***n*** = 321)		Multivariable^**a**^ (***n*** = 277)	
Variable	HR (95% CI)	*P* value	HR (95% CI)	*P* value
TAM vs. control in TILs < 50%	0.63 (0.47–0.84)	0.002	0.60 (0.43–0.83)	0.002
TAM vs. control in TILs ≥ 50%	0.84 (0.24–2.86)	0.77	0.90 (0.22–3.64)	0.88
Interaction TILs × TAM (HR ratio)	0.75 (0.21–2.65)	0.65	0.67 (0.16–2.83)	0.59

Separate effects of tamoxifen in the two TIL groups were estimated by changing the reference group for TILs in the Cox model with main effects for treatment and TILs and an interaction effect. The HR for interaction (0.75) is the ratio between the tamoxifen effects in low and high TILs, i.e. 0.63/0.84. All analyses were stratified by study region

*Abbreviations*: *ER* oestrogen receptor, *CI* confidence interval, *HR* hazard ratio, *TAM* tamoxifen, *TILs* tumour-infiltrating lymphocytes

^a^The following variables were included in the multivariable analysis: age (≥ 40 vs. < 40 years), nodal status (0 vs.1–3 vs. ≥ 4), tumour size (> 20 mm vs. ≤ 20 mm), histological grade (1 vs. 2 vs. 3), ER (positive vs. negative), PR (positive vs. negative), HER2 (positive vs. negative) and LVI (present vs. absent)

## Discussion

In this study, we showed that high TILs is associated with a relative reduction of the incidence of invasive BC-related events by 60% after approximately 30 years of follow-up. Interestingly, similar results were observed across BC subtypes including patients with ER+/HER2− tumours. Despite the association of high TILs with characteristics that typically indicate poor prognosis, the positive prognostic value of high TIL infiltration was retained in multivariable analysis, thereby emphasising TILs as an important independent long-term favourable prognostic factor. In addition, we present a possible predictive value of TILs on TAM benefit in patients with ER+ tumours, indicating that high infiltration of TILs might be linked to endocrine-resistant tumours.

Our results on the prognostic association of TILs in TNBC and HER2+ subtypes are in line with previous results [3, 8, 9, 11]. The associations of high TILs and negative hormone receptor status, higher NHG and higher Ki67 emphasise a higher immune infiltration in TNBC tumours. In the St. Gallen guidelines 2019, the panel recommended that TILs should be routinely characterised in TNBC tumours due to their prognostic value; however, there was not enough data on TILs to guide the use of neoadjuvant and adjuvant treatment [19]. Our data support the prognostic value of TILs in TNBC, and interestingly, our stratification of subtypes revealed similar results in patients with ER+/HER2− tumours. Previous prognostic study designs of TILs in ER+/HER2− tumours are not consistent and often based on trials of adjuvant chemo-endocrine treatment as well as neoadjuvant studies [3, 6, 7]. In a meta-analysis by Denkert et al. that included six neoadjuvant chemotherapy studies with pre-and postmenopausal women, patients with ER+/HER2− tumours and low level of TILs had an improved OS after 10 years, also after adjusting for pCR [6]. Our results show an association of high immune infiltration and better prognosis in the ER+/HER2− subgroup, which has not been reported in other adjuvant studies assessing TILs [3, 7, 11].

Previous studies of the predictive value of TILs in ER+ tumours mainly included chemotherapy trials and showed no predictive effect on either anthracycline therapy or additional taxane treatment [3, 11]. In the present study, we observed a TAM benefit regarding BCFi in patients with ER+ tumours and non-LPBC tumours (TILs < 50%), while no effect was shown in patients with LPBC tumours (TILs ≥ 50%). However, this study was not powered to detect any interaction effect between TILs and TAM, and hence, we could not demonstrate any treatment interaction. A few studies have reported findings in contrast with our results. In a study of 563 postmenopausal patients randomised to TAM or no adjuvant therapy, patients with low levels of CD8+ TILs did not seem to have any TAM benefit [28]. Dietci et al. found a higher, but not significant, Ki67 suppression after neoadjuvant endocrine therapy in the high (≥ 10%) TIL subgroup [15]. This indicated a better effect of endocrine therapy in these trials for patients with abundance of immune infiltration.

In this study, high TILs were associated with better prognosis and also co-variables indicating worse prognosis (high NHG, negative hormone receptor status, high proliferation). These findings highlight LPBC as an independent favourable prognostic factor. Importantly, LPBC was also of long-term prognostic relevance even after three decades of follow-up. The selection of study participants, shorter follow-up and the low proportion of LPBC in ER+/HER2− tumours are putative explanations to the divergent study results in this particular subtype. The distribution of subsets of the immune cell population, such as CD8+, T regulatory cells and macrophages, could be an additional explanatory factor that was not addressed in our study. The favourable outcome for patients with ER+/LPBC tumours did not seem to be further improved by TAM treatment. The relation of immune infiltration and tumour mutational burden is thought to be associated with breast cancer outcomes [29], and one hypothesis is that the tumour mutational load explains a possible endocrine resistance noted in the high TIL subgroup.

The strengths of this study are that it was based on a randomised controlled trial and included only premenopausal patients with almost 30 years of follow-up data. The results are important both due to the long-term risk of BC-related events for patients with ER+/HER2− tumours [17] and for the younger patient category. Moreover, the treatment arm consisted of only 2 years of adjuvant TAM and the patients in the control arm received no systemic therapy. Despite it has been more than three decades since the start of the SBII:2pre trial, TAM is still the adjuvant endocrine drug of choice for most premenopausal women and our data are therefore of interest for contemporary patients. The scoring of general TILs was performed by a BC pathologist on whole tumour sections, rather than tissue microarrays, according to published standard methodology [5].

The study limitations include the sample size of subgroup analyses, especially in the prognostic analyses of ER+/HER2−/LPBC tumours. TIL category was not evaluated as a continuous variable, nor did we determine TIL phenotype classifications. However, the application of TILs as a categorised variable is in line with other studies [6]. The cut-off (50%) in the interaction analysis was based upon a visual determination from the predictive cumulative incidence curves, and the cut-off level could thus be considered data-driven. The focus of this study was on the ER+/HER2− subgroup, and because of the incomplete estimations of Ki67 and PR, we were not able to distinguish between Luminal A and B-like tumours.

According to current guidelines, many patients included in the present cohort would today be treated with chemo-endocrine therapy and some patients would also be offered a gonadotropin-releasing hormone analogue [19, 20]. The possibility to de-escalate chemotherapy treatment by gene expression analyses is currently recommended as an option for some women [30, 31]. Our data, indicating prolonged BC-specific survival for patients with ER+/HER2− and LPBC tumours, might aid in identifying patients with excellent long-term prognosis for which reduced use of adjuvant chemotherapy could be considered. TILs as a predictive marker for endocrine benefit for premenopausal patients would also be desirable, and the association of TILs and outcomes may furthermore be dependent on the endocrine treatment option [32]. However, larger studies are warranted to examine the predictive effect of TILs for TAM efficacy as well as for other drugs including immunotherapy in this particular subgroup of patients. Moreover, the genomic analysis of primary tumour tissue including mutational load from the SBII:2pre cohort, in relation to TILs and outcomes, is an interesting future research topic.

## Conclusions

High immune cell infiltration was independently associated with prolonged BCFi in premenopausal patients allocated to no systemic therapy in a randomised trial. The finding was extended to comprise all BC subtypes after nearly three decades of follow-up. Furthermore, adjuvant TAM was beneficial in patients with ER+/non-LPBC tumours, but the predictive effect of TILs could not be confirmed.

## Supplementary Information

**Additional file 1. **Patient and tumour characteristics of study cohort (*n* = 477) and for the excluded patients with no scored TILs (*n* = 60).

## Data Availability

The datasets generated and/or analysed during the current study are available from the corresponding author on reasonable request.

## Abbreviations

BC: Breast cancer
BCFi: Breast cancer-free interval
CI: Confidence interval
DCIS: Ductal carcinoma in situ
ER: Oestrogen receptor
HER2: Human epidermal growth factor receptor 2
HR: Hazard ratio
IHC: Immunohistochemistry
ISH: In situ hybridization
LVI: Lymphovascular invasion
LPBC: Lymphocyte-predominant breast cancer
NHG: Nottingham histological grade
OS: Overall survival
pCR: Pathological complete response
PR: Progesterone receptor
TAM: Tamoxifen
TILs: Tumour-infiltrating lymphocytes
TNBC: Triple-negative breast cancer
UICC: Union for International Cancer Control
