# Supplementary material for: Tumour-infiltrating lymphocytes as a prognostic and tamoxifen predictive marker in premenopausal breast cancer: data from a randomised trial with long-term follow-up
Source: Breast Cancer Res. 2020 Dec 23;22:140. doi: 10.1186/s13058-020-01364-w (PMC7758933; doi:10.1186/s13058-020-01364-w)
Supplement: Supplementary file 1 — Additional file 1. Patient and tumour characteristics of study cohort (n = 477) and for the excluded patients with no scored TILs (n = 60). [file 13058_2020_1364_MOESM1_ESM.docx]

| **Additional file 1.**  Patient and tumour characteristics of study cohort (n = 477) and for the excluded patients with no scored TILs (n = 60) | | | | |
| --- | --- | --- | --- | --- |
| **Characteristics** | **TAM-treated group, n (%)** | | **Control group, n (%)** | |
|  | **Scored TILs (n = 230)** | **No scored TILs (n = 32)** | **Scored TILs (n = 247)** | **No scored TILs (n = 28)** |
| Follow-up BCFi^a^, years  Median  Range (10th and 90th percentiles) | 28  25 and 30 | 27  25 and 30 | 28  26 and 30 | 27  25 and 30^b^ |
| Age (years)  Median  Range  <40  ≥40 | 45  26–57  43 (19)  187 (81) | 47  31–55  6 (19)  26 (81) | 45  27–58  51 (21)  196 (79) | 46  29–55  5 (18)  23 (82) |
| Nodal status  Median number of positive nodes  Range  0  1–3  ≥4  Missing | 1  0–21  71 (31)  112 (49)  46 (20)  1 | 2  0–12  8 (25)  17 (53)  7 (22)  0 | 1  0–22  66 (27)  121 (49)  59 (24)  1 | 2  0–22  8 (29)  13 (46)  7 (25)  0 |
| Tumour size (mm)  Median  Range  ≤20  >20  Missing | 25  8–75  70 (31)  159 (69)  1 | 25  10–60  12 (38)  20 (63)  0 | 23  2–50  99 (40)  148 (60)  0 | 21  9–49  13 (46)  15 (54)  0 |
| Histological grade (NHG)  1  2  3  Missing | 23 (11)  90 (42)  103 (48)  14 | 4 (12)  10 (39)  13 (50)  6 | 28 (12)  102 (43)  108 (45)  9 | 2 (10)  12 (57)  7 (33)  7 |
| ER  Negative  Positive | 80 (35)  150 (65) | 11 (34)  21 (66) | 76 (31)  171 (69) | 8 (29)  20 (71) |
| PR  Negative  Positive  Missing | 78 (34)  150 (66)  2 | 11 (36)  20 (65)  1 | 76 (31)  170 (69)  1 | 11 (39)  17 (61)  0 |
| HER2  Negative  Positive  Missing | 175 (86)  28 (14)  27 | 16 (89)  2 (11)  14 | 189 (83)  38 (17)  20 | 13 (100)  0  15 |
| LVI  Absent  Present  Missing | 121 (53)  109 (47)  0 | 0  0  32 | 137 (56)  108 (44)  2 | 0  0  28 |
| Ki67 (%)  ≤10  11–25  ≥ 26  Missing | 76 (40)  61 (32)  52 (28)  41 | 7 (47)  5 (33)  3 (20)  17 | 102 (49)  46 (22)  59 (29)  40 | 7 (58)  4 (33)  1 (8)  16 |
| Histopathological type  Ductal/NST  Lobular  Medullary  Other  Missing | 182 (84)  17 (8)  10 (5)  8 (4)  13 | 14 (74)  2 (11)  1 (5)  2 (11)  13 | 200 (85)  19 (8)  13 (6)  4 (2)  11 | 8 (67)  3 (25)  1 (8)  0  16 |
| Subtype  ER+/HER2–  HER2+  TNBC  ER–/PR+  Missing | 120 (59)  28 (14)  48 (24)  7 (3)  27 | 12 (67)  2 (11)  2 (11)  2 (11)  14 | 137 (60)  38 (17)  47 (21)  5 (2)  20 | 11 (85)  0 (0)  1 (8)  1 (8)  15 |
| TILs (%)  <10  10–49  50–74  ≥75  Missing | 120 (52)  72 (31)  31 (14)  7 (3)  0 | 0  0  0  0  32 | 128 (52)  85 (34)  27 (11)  7 (3)  0 | 0  0  0  0  28 |
| Adjuvant chemotherapy and/or goserelin  No  Yes  Missing | 224 (99)  3 (1)  3 | 32 (100)  0  0 | 239 (98)  4 (2)  4 | 27 (100)  0  1 |
| Adjuvant radiotherapy  No  Yes  Missing | 38 (20)  154 (80)  38 | 5 (19)  21 (81)  6 | 37 (18)  172 (82)  38 | 0  20 (100)  8 |
| ^a^Patients without event at last follow-up  ^b^75th percentile  *Abbreviations*: *BCFi* breast cancer free-interval, *ER* oestrogen receptor, *HER2* human epidermal growth factor receptor 2, *LVI* lymphovascular invasion, *NHG* Nottingham histological grade, *NST* no special type, *PR* progesterone receptor, *TAM* tamoxifen, *TILs* tumour infiltrating lymphocytes, *TNBC* triple-negative breast cancer | | | | |
